# Identification of Protein Biomarker Signatures for Acute Myeloid Leukemia (AML) Using Both Nontargeted and Targeted Approaches

**DOI:** 10.3390/proteomes9040042

**Published:** 2021-10-30

**Authors:** Paul Dowling, Ciara Tierney, Katie Dunphy, Juho J. Miettinen, Caroline A. Heckman, Despina Bazou, Peter O’Gorman

**Affiliations:** 1 Department of Biology, Maynooth University, W23 F2H6 Maynooth, Ireland; ciara.tierney.2010@mumail.ie (C.T.); katie.dunphy.2015@mumail.ie (K.D.); 2 Institute for Molecular Medicine Finland—FIMM, HiLIFE—Helsinki Institute of Life Science, iCAN Digital Cancer Medicine Flagship, University of Helsinki, FI-00014 Helsinki, Finland; juho.miettinen@helsinki.fi (J.J.M.); caroline.heckman@helsinki.fi (C.A.H.); 3 Department of Haematology, Mater Misericordiae University Hospital, D07 KH4C Dublin, Ireland; despina.bazou@ucd.ie (D.B.); pogorman@mirtireland.com (P.O.)

**Keywords:** acute myeloid leukemia, biomarkers, immunoassay, mass spectrometry, proteomics

## Abstract

Acute myeloid leukemia (AML) is characterized by an increasing number of clonal myeloid blast cells which are incapable of differentiating into mature leukocytes. AML risk stratification is based on genetic background, which also serves as a means to identify the optimal treatment of individual patients. However, constant refinements are needed, and the inclusion of significant measurements, based on the various omics approaches that are currently available to researchers/clinicians, have the potential to increase overall accuracy with respect to patient management. Using both nontargeted (label-free mass spectrometry) and targeted (multiplex immunoassays) proteomics, a range of proteins were found to be significantly changed in AML patients with different genetic backgrounds. The inclusion of validated proteomic biomarker panels could be an important factor in the prognostic classification of AML patients. The ability to measure both cellular and secreted analytes, at diagnosis and during the course of treatment, has advantages in identifying transforming biological mechanisms in patients, assisting important clinical management decisions.

## 1. Introduction

Acute myeloid leukemia (AML) is a highly heterogenous disease of the blood and bone marrow, characterized by the uncontrolled proliferation of cells from the myeloid lineage [1]. AML is the second most common form of leukemia in adults, and accounts for approximately 1% of new cancer diagnoses [2]. The majority of patients, following intensive chemotherapy, achieve complete remission; however, AML remains a highly fatal disease, with a disheartening five-year survival rate of ~24% [3]. The high fatality of this disease is attributed to the presence of primary resistance in a subset of patients and a high relapse rate with therapy-resistant disease following complete remission [4].

According to the French-American-British (FAB) system, there are eight types of AML (M0–M7), each with precise morphological characteristics and differentiation stages [5]. The more recent World Health Organization (WHO) classification considers clinical features, morphology, immunophenotyping, cytogenetics, and molecular genetics [6].

Cytogenetic abnormalities, including chromosomal translocations, deletions, and duplications, in addition to molecular mutations, such as mutations in the nucleophosmin-1 (*NPM-1*) or FMS-like tyrosine kinase 3 (FLT3) genes, are powerful prognostic markers in AML. In 2017, European LeukemiaNet (ELN) published revised recommendations to aid the interpretation of genetic abnormalities for risk stratification in AML [7,8]. The ELN recommendations stratify patients into three prognostic categories; “favorable”, “intermediate” or “adverse”, based on the genetic profile of patients. These recommendations are used to determine the risk of chemoresistance, a key factor in determining the best course of treatment for patients, as the benefit of highly intensive chemotherapy may outweigh the potential therapy-induced toxicities in patients with “intermediate” or “adverse” risk [8,9].

Despite many clinicians adopting this method of risk stratification, the crucial factors involved in relapse and chemoresistance have yet to be identified, with several studies proposing additional prognostic markers which may improve the prognostic value of the ELN recommendations [10,11,12]. Furthermore, the risk of relapse with therapy-resistant disease in patients with “favorable” risk remains relatively high, i.e., occurring in 30–35% of cases [8]. The high recurrence rate and aggressiveness of this disease heightens the need for an expansion of current biomarkers to improve prognostic classification, risk assessment and therapeutic decision-making in AML.

Advances in high-throughput proteomic techniques, especially in the area of mass spectrometry, has pushed proteomics to the forefront of current efforts in the discovery of novel clinically relevant biomarkers [13]. Analyzing the proteomic profile of AML patients has led to the identification of various potential therapeutic targets and candidate protein biomarkers to predict AML relapse and therapeutic efficiency [14,15,16,17].

In this study, we compared the proteomic profiles of clinical AML cells from favorable (group 1), intermediate (group 2) and adverse (group 3) risk patients based on the ELN recommendations to identify specific proteins associated with each risk group. Further proteomic analyses of the matched AML serum samples were conducted to identify and quantify cytokine levels between the three risk groups. The proteomic profiling of the ELN based risk groups facilitated the identification of differential protein levels between risk groups, thus, improving our current understanding of AML prognosis and identifying potential biomarkers to aid prognostic classifications in AML.

## 2. Materials and Methods

### 2.1. Clinical Samples

Matching Peripheral Blood (PB) and bone marrow (BM) patient samples were collected at the same time after receipt of written informed consent from the Helsinki University Hospital Comprehensive Cancer Center (Helsinki University Hospital Ethics Committee, decision number 3613/06.01.05.01.00/2014) and in compliance with the Declaration of Helsinki. In total, 41 samples from AML patients were collected. The patient characteristics are presented in Table 1. Cytogenetic and molecular genetic data were available for all patients, and genetic group (favorable, intermediate, and adverse) was defined according to the European LeukemiaNet. Mononuclear cells (MNCs) were isolated from the BM samples by Ficoll density gradient centrifugation (GE Healthcare, Little Chalfont, UK). MNCs were viably cryopreserved in 10% DMSO and 90% fetal calf serum until further analysis.

### 2.2. Sample Preparation for Mass Spectrometry

BM aspirate cells were lysed in an SDT-lysis buffer (4% SDS, 100 mM Tris/HCl, 100 mM DTT, pH 7.6) using 1:10 sample to buffer ratio and heated at 95 °C for 3–5 min. DNA was sheared by sonication to reduce the viscosity of the sample. Before starting sample processing, the lysate was clarified by centrifugation at 16,000× *g* for 5 min. A Pierce 660 nm protein assay system was used to determine protein concentration. Suspensions were then buffer exchanged using the filter-aided sample preparation (FASP) method in a buffer containing 8 M urea/50 mM NH_4_HCO_3_/0.1% ProteaseMax. After reduction with dithiothreitol and iodoacetic acid-mediated alkylation, a double digestion was performed using Lys-C (for 4 h at 37 °C) and trypsin (overnight at 37 °C) on 5 μg of BM aspirate protein. Digested samples were desalted prior to analysis using C18 spin columns (ThermoFisher Scientific, Hemel Hempstead, UK), dried through vacuum centrifugation and resuspended in mass spectrometry loading buffer (2% acetonitrile (ACN), 0.05% trifluoroacetic acid (TFA) in LC-MS grade water). Peptides were vortexed, sonicated and briefly centrifuged at 14,000× *g* and the supernatant transferred to mass spectrometry vials for label-free liquid chromatography mass spectrometry (LC-MS/MS).

### 2.3. Label-Free Liquid Chromatography Mass Spectrometry

First, 500 ng of each digested sample was loaded onto a Q-Exactive high-resolution accurate mass spectrometer connected to a Dionex Ultimate 3000 (RSLCnano) chromatography system (ThermoFisher Scientific, Hemel Hempstead, UK). Sample loading was carried out by an auto-sampler onto a C18 trap column (C18 PepMap, 300 μm id × 5 mm, 5 μm particle size, 100 Å pore size; Thermo Fisher Scientific). The trap column was switched on-line with an analytical Biobasic C18 Picofrit column (C18 PepMap, 75 μm id × 50 cm, 2 μm particle size, 100 Å pore size: Dionex). Peptides were eluted over a 65 min binary gradient [solvent A: 2% (*v*/*v*) ACN and 0.1% (*v*/*v*) formic acid in LC-MS grade water and solvent B: 80% (*v*/*v*) ACN and 0.1% (*v*/*v*) formic acid in LC-MS grade water]: 3% solvent B for 5 min, 3–10% solvent B for 5 min, 10–40% solvent B for 30 min, 40–90% solvent B for 5 min, 90% solvent B for 5 min and 3% solvent B for 10 min. The column flow rate was set to 0.3 μL/min. Data were acquired with Xcalibur software (Thermo Fisher Scientific). The mass spectrometer was externally calibrated and operated in positive, data-dependent mode. A full survey MS scan was performed in the 300–1700 *m*/*z* range with a resolution of 140,000 (*m*/*z* 200) and a lock mass of 445.12003. Collision-induced dissociation (CID) fragmentation was carried out with the fifteen most intense ions per scan and at 17,500 resolution. Within 30 s a dynamic exclusion window was applied. An isolation window of 2 *m*/*z* and one microscan were used to collect suitable tandem mass spectra.

### 2.4. Protein Identification and Quantification

Data analysis, processing and visualization for urine protein identification and label-free quantification (LFQ) normalization of MS/MS data was performed using MaxQuant v1.5.2.8 (http://www.maxquant.org) (accessed on 4 March 2019) and Perseus v.1.5.6.0 (www.maxquant.org/) (accessed on 8 March 2019) software. Differential protein expression patterns in the AML (favorable, intermediate, and adverse prognostic groups) proteomes were initially identified using MaxQuant software and the Andromeda search engine to explore the detected features against the UniProtKB/SwissProt database for Homo sapien. The following search parameters were used: (i) first search peptide tolerance of 20 ppm, (ii) main search peptide tolerance of 4.5 ppm, (iii) cysteine carbamidomethylation set as a fixed modification, (iv) methionine oxidation set as a variable modification, (v) a maximum of two missed cleavage sites and (vi) a minimum peptide length of seven amino acids. The false discovery rate (FDR) was set to 1% for both peptides and proteins using a target-decoy approach. Relative quantification was performed using the MaxLFQ algorithm. The “proteinGroups.txt” file produced by MaxQuant was further analyzed in Perseus. Proteins that matched to the reverse database or a contaminants database or that were only identified by site were removed. The LFQ intensities were log2 transformed, and only proteins found in all seven replicates in at least one group were used for further analysis. Data imputation was performed to replace missing values with values that simulate signals from peptides with low abundance chosen from a normal distribution specified by a downshift of 1.8 times the mean standard deviation of all measured values and a width of 0.3 times this standard deviation. A two-sample *t*-test was performed using *p* < 0.05 on data post imputation, to identify statistically significant differentially abundant proteins. The freely available software package PANTHER (http://pantherdb.org/) (accessed on 4 April 2019), was used to identify protein classes and characterize potential protein interactions, respectively. Statistical overrepresentation tests (PANTHER GO-Pathway) of protein sets were performed using the PANTHER database (http://PANTHERdb.org/) (accessed on 6 April 2019). Protein lists were uploaded in gene symbol format, and default whole genome lists from the appropriate species were used as reference [18]. To analyze statistical significance, Fisher’s exact test with Benjamini–Hochberg False Discovery Rate correction (FDR) was applied [19].

### 2.5. Luminex Assay

Blood samples were evaluated using panels of analytes on the Luminex xMAP technology bead-based multiplexed immunoassay system. Panel 1 (L1CAM, CA9, Mesothelin, Midkine, Hepsin, Kallikrein 6, TGM2, ALDH1A1, EpCAM and CD44). Panel 2 (EGF, Eotaxin, G-CSF, GM-CSF, IFNα2, IFNγ, IL-10, IL-12p40, IL-12p70, IL-13, IL-15, IL-17A, IL-1RA, IL-1α, IL-1β, IL-2, IL-3, IL-4, IL-5, IL-6, IL-7, IL-8, IP-10, MCP-1, MIP-1α, MIP-1β, TNFα, TNFβ and VEGF). Panel 3 (eotaxin-2, MCP-2, BCA-1, MCP-4, I-309, IL-16, TARC, 6CKine, eotaxin-3, LIF, TPO, SCF, TSLP, IL-33, IL-20, IL-21, IL-23, TRAIL, CTACK, SDF-1α+β, ENA-78, MIP-1d and IL-28A). Samples were run in duplicate, along with blanks, standards, and high and low concentration controls. Fluorescent values beyond the range of the standards were extrapolated (unless the fluorescence intensity was below that of the blanks). Average value of duplicates was used for data analysis.

## 3. Results

### 3.1. Proteomics Profiling of Human Bone Marrow Cells

Proteomic profiles on bone marrow aspirates from “favorable”, “intermediate” and “adverse” risk AML patients were generated using label-free mass spectrometry. Label-free mass spectrometry was selected as the quantitative approach because of the minimal sample processing necessary to achieve relative quantification (Supplementary Tables S1–S3). Bone marrow samples of high quality from 41 human subjects, 15 males and 26 females, were available for proteomics analysis. Their age ranged from 16.5 to 78.1 years with a median of 57 years (Table 1). Group 1 had male (3), female (8) with an average diagnostic age of 48.3; Group 2, male (6), female (10) with an average diagnostic age of 57.5 and Group 3, male (6), female (8) with an average diagnostic age of 54.3.

Comparing group 1 (favorable) to group 2 (intermediate), 18 proteins were found to be significantly changed, with CAH1 (Carbonic anhydrase 1), 5.2-fold elevated in group 2 (*p* = 0.035) representing the largest abundance difference between these groups (Table 2; Supplementary Tables S5–S7). Comparing group 2 to group 3 (adverse), 41 proteins were found to be significantly changed, with PRKDC (protein kinase, DNA-activated, catalytic subunit), 12-fold elevated in group 2 (*p* = 0.010), representing the largest abundance difference between these groups. The highest number of statistically significant proteins was found when comparing group 1 to group 3. In total, 64 proteins were determined to be significantly changed between these groups, with TIF1B 7.0-fold elevated in group 3 (*p* = 0.027).

### 3.2. Pathway Analysis

**Figure 1:**
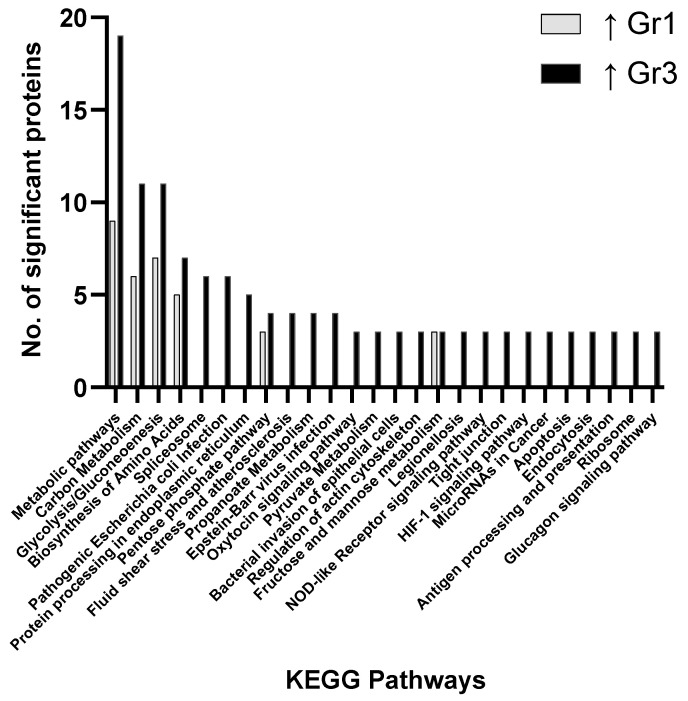
Pathway analysis (cells) comparing significant proteins found to be elevated in Group 1 (Gr1—favourable) and Group 3 (Gr3—adverse) when compared.

**Figure 2:**
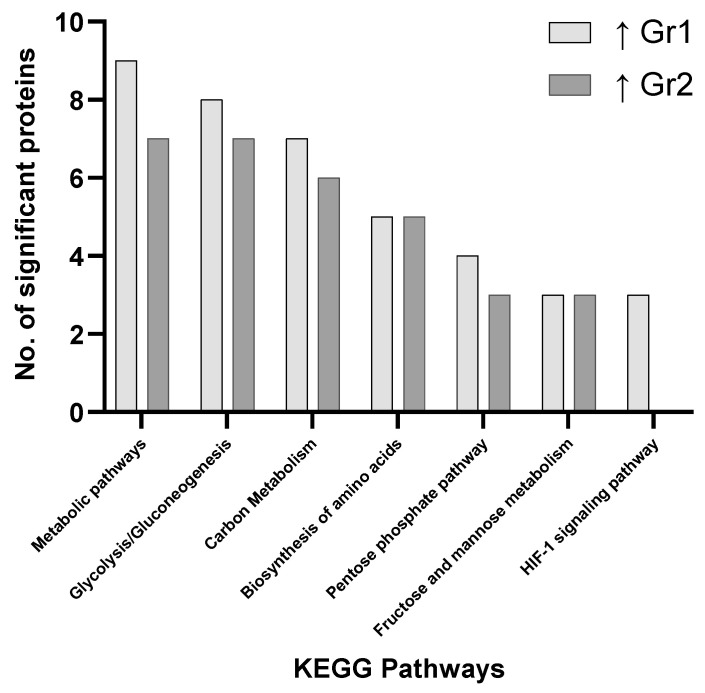
Pathway analysis (cells) comparing significant proteins found to be elevated in Group 1 (Gr1—favorable) and Group 2 (Gr2—intermediate) when compared.

**Figure 3:**
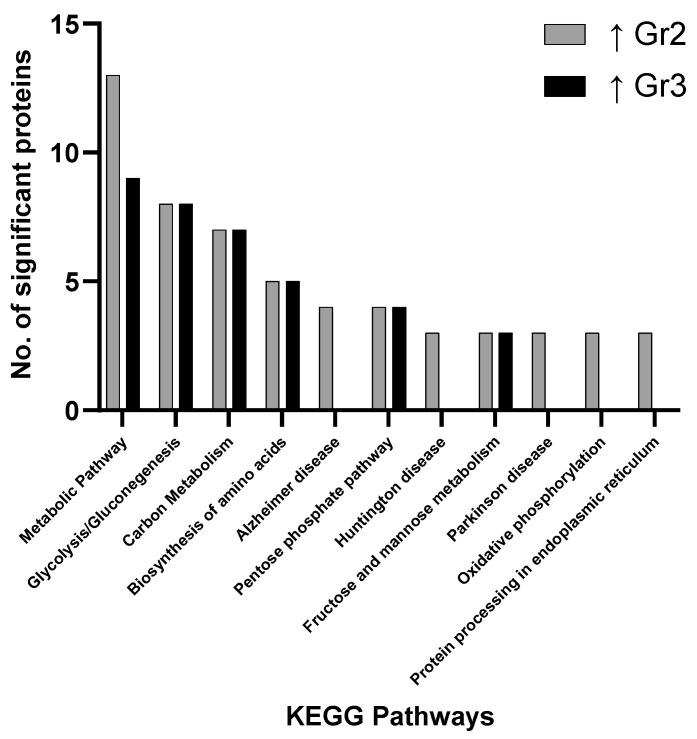
Pathway analysis (cells) comparing significant proteins found to be elevated in Group 2 (Gr2—intermediate) and Group 3 (Gr3—adverse) when compared.

Focusing on the group 1 vs. group 3 comparison, more proteins associated with metabolic pathways, carbon metabolism, glycolysis/gluconeogenesis and biosynthesis of amino acids were discovered to be elevated in group 3 (Figure 1). Of the proteins identified as significantly changed between group 1 and group 3, those involved in propanoate metabolism, oxytocin signaling pathway, pyruvate metabolism, regulation of the actin cytoskeleton, nucleotide-binding oligomerization domain (NOD)-like receptor signaling pathway, tight junctions, hypoxia-inducible factor 1 (HIF-1) signaling pathway, apoptosis and the glucagon signaling pathway were uniquely associated with group 3. Overall, the majority of proteins were found to be significantly elevated in group 3 compared to group 1 (56 proteins vs. 8 proteins) (Figure 1, Figure 2 and Figure 3).

The main pathways involving significantly changed proteins when evaluating all groups focus on metabolic pathways, carbon metabolism, glycolysis/gluconeogenesis, biosynthesis of amino acids, the pentose phosphate pathway, pyruvate metabolism and fructose/mannose metabolism. These results indicate how primary AML samples show variations in distinct metabolic pathway and the associated prognostic impact this may have on clinical outcome (Figure 1, Figure 2 and Figure 3). No pathways were found to be significant based on pathway enrichment analysis filtered by FDR-adjusted *p*-value (<0.05) using the PANTHER Classification System because of the small protein lists submitted.

### 3.3. Targeted Proteomics Analysis

The simultaneous measurement of different analytes from a single sample is an emerging area for achieving efficient and high-throughput detection in several applications, including biomarker discovery. In this analysis, the Luminex platform was employed which includes a robust workflow and overcomes sample limitation problems, thus eliminating the need to perform parallel individual measurements using single-plex approaches. Using antibody coated magnetic beads, panels evaluating human circulating cancer biomarkers and human cytokine/chemokine/growth factors were run and subsequently using xPONENT, standard curve fitting models for quantitative analysis were generated (Supplementary Table S4).

**Figure 4:**
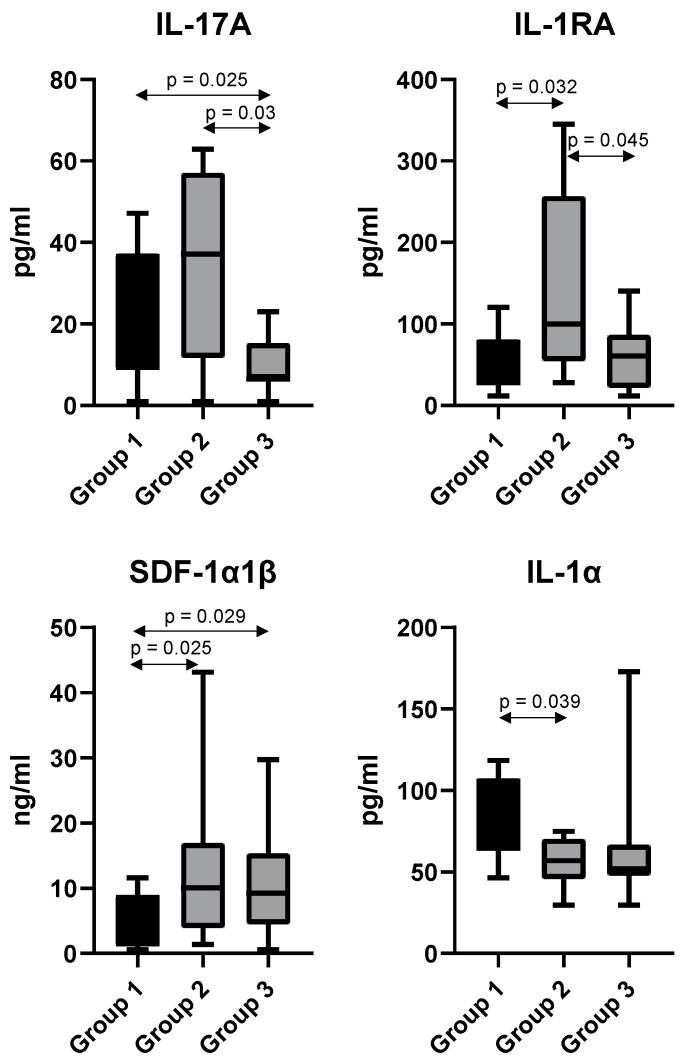
Box and Whisker plots for IL-17A, IL-1RA, SDF-1α1β and IL-1α based on results from serum biomarker analysis using targeted multiplexed immunoassays.

Serum samples from 41 human subjects were analyzed, with IL-17A, IL-1RA, IL-1α and SDF-1α1β found to be significantly changed in abundance across the different risk groups (Figure 4). IL-17A was found to have median values of 30 pg/mL, 37 pg/mL, and 6 pg/mL; IL-1RA 27 pg/mL, 99 pg/mL, and 60 pg/mL; IL-1α 85 pg/mL, 56 pg/mL, and 52 pg/mL; SDF-1α1β 2 ng/mL, 10 ng/mL, and 9 ng/mL in groups 1–3 respectively. In terms of fold-changes for these analytes between the 3 groups, the most significant changes related to IL-17A, with a 5-fold and 6.2-fold decrease in abundance observed when comparing group 3 with groups 1 and 2 respectively. SDF-1α1β was determined to have a 5-fold and 4.5-fold increase in abundance in group 2 and group 3 respectively, when compared to group 1.

## 4. Discussion

In this study, we used quantitative proteomic techniques to identify specific proteins associated with a “favorable”, “intermediate” or “adverse” prognosis. We identified elevated levels of metabolic-related proteins in AML cells from patients with an “adverse” prognosis when compared with AML cells from patients with a “favorable” prognosis. A change in the levels of several cytokines (IL-17A, IL-1RA, IL-1α and SDF-1α1β) between the risk groups was also identified in matched AML serum samples.

The complexity and heterogeneity of AML is illustrated by its classification into different disease subtypes based on distinct differences in genetic make-up, morphology and clinical presentation of AML [1]. Furthermore, AML patients often display intra-tumoral heterogeneity with molecularly distinct subclones, often present at a low frequency, enhancing the difficulties associated with efficient biomarker discovery and limits the efficacy of target-specific drugs [20,21,22,23]. To combat the heterogeneity of this disease, extensive research is required to boost current efforts in biomarker discovery to facilitate more accurate prognostic classifications and better therapeutic decisions.

Altered metabolism is a well-known hallmark of cancer associated with the reprogramming of metabolic activities to support a number of pro-anabolic pathways promoting tumorigenesis and disease progression [24]. Metabolomics has emerged as an important -omics technology in identifying novel biomarkers and therapeutic targets. Several studies have reported dysregulation in metabolic pathways in AML [25,26,27,28,29,30]. Mutations in the gene encoding the isocitrate dehydrogenase (IDH) enzyme involved in the tricarboxylic acid (TCA) cycle results in the production of the oncometabolite 2-hydroxyglutarate (2HG) and has been identified in ~6% of AML patients [31]. Analysis of AML patient serum revealed 2HG levels correlate with IDH mutational status and suggest a potential role as a prognostic, predictive and therapeutic-monitoring biomarker in AML [32,33]. A mass-spectrometry-based metabolomics study by Chen et al. identified a glucose metabolism prognostic biomarker signature consisting of 6 metabolites [34]. The broadening of metabolomics studies has resulted in various preclinical and/or clinical trials analyzing the effect of chemotherapies targeting metabolism in AML [15,35,36].

Previous studies describing dysregulated metabolism in AML correlate with our findings. Our studies revealed elevated metabolic-related protein levels in “adverse” risk versus “favorable” risk clinical AML cell lysates. A total of 10 metabolic-related proteins were elevated including peroxiredoxin-6, neutral alpha-glucosidase AB, 3-ketoacyl-CoA thiolase (3-KAT), hydroxyacyl-CoA dehydrogenase alpha (HADHA), transaldolase, cytosolic nonspecific dipeptidase, lactate dehydrogenase A and B, dihydrolipoyl dehydrogenase and nucleoside diphosphate kinase B. Several of these metabolic proteins have previously been investigated in AML.

3-KAT and HADHA enzymes make up part of the trifunctional protein complex involved in catalyzing acetyl-CoA production by β-oxidation during fatty acid oxidation (FAO) [37]. FAO was previously reported to be increased in chemo-resistant AML cells [38]. The overexpression of the FAO-related enzyme carnitine palmitoyl transferase 1A (CPT1A) was also shown to be a predictor of poor outcome in AML [39]. Furthermore, bone marrow adipocytes were found to promote acute monocytic leukemia (AMoL) survival via increased FAO, determined by detecting increasing levels of HADHA following AMoL co-culturing [40]. The FAO inhibitor, Avocatin B, was also found to possess potent anti-AML activity [41]. When combined, these studies implicate increased FAO and elevated FAO-related protein levels in the pathogenesis of AML.

Lactate dehydrogenase (LDH) is a well-established biomarker capable of aiding prognosis in a variety of cancers including AML [42,43,44,45]. Recent studies have indicated the potential of LDH as a prognostic marker in AML patients undergoing allogeneic hematopoietic stem cell transplantation (HSCT), as well as a predictive marker of AML patient outcome following sibling bone marrow transplant (BMT) [46,47]. Dihydrolipoyl dehydrogenase is a catalytic subunit of the pyruvate dehydrogenase complex, a target of the lipoate mimetic compound CPI-613 currently being evaluated in a Phase 3 clinical trial in combination with other approved chemotherapeutics for the treatment of relapsed or refractory AML [48]. Low expression levels of nm23-H2, also known as nucleoside diphosphate kinase B, was previously reported to be a good prognostic biomarker in AML [49]. Similarly, the overexpression of nm23-H2 was found to be associated with the “adverse” risk group in our study.

Research in the field of oncometabolism has revealed certain metabolic alterations as key contributors to tumorigenesis and tumor progression [50]. In AML, alterations in several metabolic pathways such as the production of reactive oxygen species (ROS) and increased levels of oxidative phosphorylation, have been linked to clinicopathological features of AML including aggressive disease and chemoresistance [38,51]. A recent study by Lo Presti et al. highlighted the strong influence of metabolic reprogramming on AML prognosis by identifying distinct changes in the metabolic profile of leukemic cells according to their mutational profile and stage of differentiation [52].

Future validation studies, a limitation of this study, on these elevated metabolic-related proteins may lead to the development of a metabolism-based prognostic biomarker signature to boost the prognostic value of current ELN recommendations. Our results show a clear increase in the number of significantly elevated metabolic-related proteins in the “intermediate” risk group. This risk group is the largest subset with many patients displaying heterogenous outcomes, suggesting a need for further stratification [53]. Several studies have identified specific genetic mutations in “intermediate” risk patients that veer towards a more favorable outcome whereas others indicate a poor outcome [54,55]. Thus, further analysis of the dysregulated metabolic-related proteins identified in the “intermediate” risk group and the survival outcome of the patients analyzed may reveal interesting patterns corresponding patient prognosis.

The assessment of bone marrow remains the “gold standard” for the diagnosis and monitoring of AML following treatment. Bone marrow biopsies are invasive and painful procedures; therefore, considerable efforts are being made to develop less invasive means of disease diagnosis and monitoring [56,57]. Analysis of AML patient serum provides an easier and less intrusive method of identifying biomarkers. Our multiplex assays focused on identifying altered chemokine/cytokine levels in AML serum samples matched to the previously analyzed AML cells. Four interesting cytokines (IL-1RA, IL-1α, IL-17A, SDF-1α1β) were found to be significantly dysregulated between single or multiple risk groups.

Interleukin-1 receptor antagonist (IL-1RA) is an anti-inflammatory cytokine that competitively binds to the IL-1 receptor (IL-1R), inhibiting binding of IL-1α and IL-1β and thus preventing downstream signaling cascade initiation [58]. Heterogeneous results from previous studies imply fluctuating levels of IL-1RA in AML patient sera [59,60,61]. One study reported increased, unchanged, and decreased levels of AML proliferation following exposure to IL-1RA, which causes further confusion on the role of IL-1RA in AML [62]. Despite a significant increase in serum IL-1RA levels in the “intermediate” risk group, additional studies are required to validate these findings.

The IL-1 cytokines, IL-1α and IL-1β, exert their pro-inflammatory effects via binding to IL-1R. IL-1α is constitutively secreted and has been reported to possess tumor-promoting or tumor-suppressing properties depending on the type of malignancy [63]. Despite, many articles focusing on IL-1β in AML, studies focusing on IL-1α are lacking [64]. We report increased serum IL-1α levels in patients with a favorable prognosis. Further studies are required to elucidate whether IL-1α plays an anti-tumorigenic or pro-tumorigenic role in AML to determine the prognostic relevance of high levels of this cytokine in “favorable” risk AML.

IL-17A is a hematopoietic stimulatory cytokine mainly secreted by T helper 17 cells. IL-17A has previously been reported to promote the proliferation of IL-17 receptor (IL-17R)-positive AML cells through the activation of proliferative signaling pathways such as JAK2/STAT3 [65]. IL-17A was found to be significantly decreased in the “adverse” risk group compared to “favorable” or “intermediate” risk groups. Despite reports that increased serum IL-17A represents a poor prognostic marker in AML, our findings may suggest an immuno-protective role of IL-17A in some cases, as described in other cancer types [66,67,68,69]. Heterogeneous and contradicting results regarding serum cytokines levels complicates the ability to accurately define these cytokines as useful biomarkers in AML. Further studies to elucidate the lone actions and crosstalk between cytokines is required to confidently identify a panel of serum cytokine biomarkers to aid disease monitoring and prognostication in AML.

Stromal cell-derived factor-1 α and β (SDF-1/CXCL12) are commonly expressed cytokines in various cells and tissues. CXCL12 binds to the CXCR4 receptor leading to the activation of intracellular events such as chemotaxis, proliferation, and transcription [70,71]. CXCR4 is expressed on almost all hematological cell types including lymphocytes and hematopoietic stem cells (HSCs). The CXCL12/CXCR4 ligand/receptor complex is associated with tumor progression, angiogenesis, metastasis, and survival in various malignancies [72,73,74]. CXCL12 levels has been shown to have prognostic significance in cancer, with high levels associated with adverse outcome in esophagogastric, pancreatic, and lung cancer; and, conversely, associated with enhanced survival in breast cancer [75].

In AML, the CXCL12/CXCR4 complex is exploited to initiate pro-survival signaling and homing of AML blasts to the protective bone marrow niche [76,77]. The frequency of bone marrow stromal cells secreting CXCL12 (CXCL12^+^) in the BM and CXCR4 expression is increased in AML, potentially due to hypoxic conditions within the microenvironment [78,79]. High CXCL12 causes a migration of CXCR4^+^ leukemic blasts towards the high CXCL12 levels within the protective bone marrow niche [77,80]. The CXCR4^+^ stromal cells and CXCR4^+^ leukemic cells create a bi-directional interaction network within the BM resulting in the constitutive activation of proliferative and survival signaling pathways [79,81]. High levels of CXCL12 is suggested to promote the retention of AML-blasts with the bone marrow microenvironment, thus, reducing the susceptibility of these blasts to chemotherapeutics [77]. Our AML serum analysis found that CXCL12 was elevated in “intermediate” and “adverse” risk AML patients. This finding corresponds with previous studies suggesting that high CXCR4 expression is indicative of poor prognosis in AML [82]. Recently, researchers revealed a new CXCR4 receptor antagonist IgG1 antibody (PF-06747143) capable of binding strongly to AML cell lines and to AML primary cells inhibiting their chemotaxis in response to CXCL12 [83]. Monitoring of serum CXCL12 levels between the “favorable” risk group and the “intermediate” and “adverse” risk groups represents a potential marker of disease progression in AML with higher levels corresponding to more adverse outcomes. Blockade of the CXCL12 pathway, using a commercially available CXCR4 antagonists such as plerixafor, may be an efficient method of modulating AML cell proliferation and chemotherapy resistance [84]. As interpatient variabilities are often seen in serum cytokine levels, further context-dependent studies in relation to AML subtypes or age will improve our understanding of changing cytokine levels in AML sera [85,86,87].

As our understanding of the pathophysiology mechanisms associated with AML increases, this has directly contributed to be generation of new therapeutic approaches to treat this malignancy. Much success has been achieved recently using inhibitors of FMS-like tyrosine kinase 3 (FLT3), isocitrate dehydrogenase (IDH) and Bcl-2 (B-cell lymphoma 2). Within the AML cohort, kinase 3 (FLT3) mutations are detected in approximately one-third of patients [88]. Sorafenib is the most common FLT3 inhibitor used, with high activity against internal tandem duplication ITD mutations instead of wild-type FLT3 and tyrosine kinase domain (TKD) mutations [89]. Mutations in IDH1 or IDH2 are detected in approximately 20% of AML patients inducing amino acid changes in conserved residues [90]. Specific IDH1 and IDH2 inhibitors include ivosidenib and enasidenib. Strategies to increase the efficacy of these inhibitors continues, for example in combination with venetoclax [91]. Indeed, venetoclax-based combinations have improved outcomes, including both remission rates and overall survival for older patients. Combinations of venetoclax, with either hypomethylating agents (HMA) or low dose cytarabine (LDAC), have shown promising results in clinical trials [92]. Glasdegib, a hedgehog pathway inhibitor and immune checkpoint inhibitors (nivolumab—PD-1 and ipilimumab—CTLA-4) are currently other therapeutic strategies that are continually being refined with respect to the most efficacious combination to include these therapeutic as part of [93,94]. In the near future, epigenetic modifiers (RMDs), microRNAs and suppressor of cytokine signaling are likely to be utilized clinically with respect to their anti-leukemia activity [95].

Many benefits exist for using a liquid biopsy for the analysis of biomarkers, with plasma, serum and saliva routinely employed. A liquid biopsy is relatively noninvasive to acquire and is generally a less time-consuming procedure than other approaches. Together with soluble proteins found in biofluids, circulating tumor cells (CTCs), circulating tumor DNA (ctDNA), and exosomes are regularly investigated because of their wide range of clinical applications. The presence of leukemic myeloblasts in peripheral blood from AML patients makes this biofluids especially attractive for the analysis of biomarkers, soluble or cellular-based. An advantage of measuring soluble proteins present in peripheral blood is that less processing of the samples needs to be completed (for examples measuring cytokine levels by enzyme-linked immunosorbent assay (ELISA)), speeding up the diagnostic/prognostic test result and ultimately providing a more cost-effective methodology.

An important consideration for this study is that identifications are based on peptides derived from canonical sequences of public databases, and subsequent validation studies will need to take into account the complexity of associated proteoforms that may be present (for example, splice variants and post-translational modifications (PTMs)), when designing these studies [96].

## 5. Conclusions

Current clinically-used biomarkers in AML are based almost solely on genomic abnormalities, as outlined in the ELN recommendations [8]. The inclusion of validated proteomic biomarkers would establish a more powerful array of factors to improve the prognostic classification of AML patients and ensure more calculated therapeutic decisions are made based on a patient’s prognosis and various other factors. Our study identified elevated metabolic-related proteins associated with adverse risk in AML, supporting evidence of the involvement of increased metabolism in AML. Several differentially expressed cytokines were also identified in the sera of patients from the three risk groups. IL-17A and IL-1RA levels were found to fluctuate across the different prognostic groups; however, both SDF-1α1β (CXCL12) and IL-1α were consistently found to increase and decrease in abundance, respectively, across the different groups. The consistent decrease in IL-1α in the different prognostic groups may point to its role as an antitumorigenic protein, as reported by others. High CXCL12 levels in intermediate and adverse risk groups were of particular interest due to the role of this chemokine in AML pathogenesis. Therefore, further efforts to validate CXCL12 as a prognostic biomarker are warranted. This study supports a role for increased CXCL12 (serum levels) and increased metabolism-related proteins in the risk profile associated with different cohorts of AML patients.

## Figures and Tables

**Table 1:** Patient Data including gender, age at diagnosis, risk classification, diagnosis type and percentage (%) blasts in bone marrow (BM). DEK-NUP214: fusion gene created by the translocation occurring between specific introns in the gene DEK on chromosome 6 and the gene NUP214 on chromosome 9. NOS: Not otherwise specified.

Sample ID	Gender	Diagnosis Age	Risk Class	Diagnosis Type	% Blasts in BM
1	Female	46.4	1	9871 Ac. myelomonocytic leuk. w abn. mar. eosinophils	50
2	Female	35.3	1	9896 Acute myeloid leukemia, t(8;21)(q22;q22)	60
3	Male	21.6	1	9896 Acute myeloid leukemia, t(8;21)(q22;q22)	40
4	Female	67.3	1	9861 Acute myeloid leukemia	75
5	Female	68.8	1	9861 Acute myeloid leukemia	n/a
6	Male	16.8	1	9896 Acute myeloid leukemia, t(8;21)(q22;q22)	26
7	Female	55.5	1	9873 Acute myeloid leukemia without maturation	90
8	Female	44.8	1	9861 Acute myeloid leukemia	65
9	Female	53.5	1	9874 Acute myeloid leukemia with maturation	20
10	Male	72.8	1	9861 Acute myeloid leukemia	n/a
11	Female	48.7	1	9891 Acute monocytic leukemia	8
12	Male	76.9	2	9861 Acute myeloid leukemia	n/a
13	Female	62.9	2	9874 Acute myeloid leukemia with maturation	30
14	Male	56.5	2	9861 Acute myeloid leukemia	23
15	Female	63.8	2	9861 Acute myeloid leukemia	70
16	Female	78.1	2	9891 Acute monocytic leukemia	60
17	Female	24.3	2	9861 Acute myeloid leukemia	63
18	Male	67.3	2	9895 Acute myeloid leuk. with multilineage dysplasia	37
19	Female	48.6	2	9873 Acute myeloid leukemia without maturation	60
20	Male	72.6	2	9874 Acute myeloid leukemia with maturation	33
21	Male	16.5	2	9891 Acute monocytic leukemia	80
22	Female	62.9	2	9861 Acute myeloid leukemia	22
23	Female	61.5	2	9891 Acute monocytic leukemia	40
24	Female	66.7	2	9897 Acute myeloid leukemia, 11q23 abnormalities	15
25	Male	57	2	9874 Acute myeloid leukemia with maturation	42
26	Female	35.4	2	9920 Therapy-related acute myeloid leukemia, NOS	95
27	Female	68.2	2	del(9q)w23	60
28	Female	76.6	3	9873 Acute myeloid leukemia without maturation	91
29	Female	54.3	3	9867 Acute myelomonocytic leukemia	12
30	Male	28.6	3	9891 Acute monocytic leukemia	45
31	Male	66.7	3	9873 Acute myeloid leukemia without maturation	85
32	Female	52	3	9896 Acute myeloid leukemia, t(8;21)(q22;q22)	91
33	Female	21.8	3	9873 Acute myeloid leukemia without maturation	79
34	Male	44.6	3	9873 Acute myeloid leukemia without maturation	73
35	Female	71.1	3	9873 Acute myeloid leukemia without maturation	70
36	Female	39.7	3	9891 Acute monocytic leukemia	40
37	Male	40.6	3	9861 Acute myeloid leukemia	85
38	Female	59.4	3	9865 Acute myeloid leukemia with t(6;9)(p23;q34) DEK-NUP214	85
39	Male	77.7	3	9895 Acute myeloid leuk. with multilineage dysplasia	16
40	Male	62.5	3	9727 Precursor cell lymphoblastic lymphoma, NOS	91
41	Female	64.7	3	9920 Therapy-related acute myeloid leukemia, NOS	65

**Table 2:** Mass spectrometry data for group 1 vs. group 2; group 2 vs. group 3 and group 2 vs. group 3 including gene name, *p*-value, and direction of fold-change.

**Group 1 vs. Group 2**			
**Gene Name**	**ANOVA *p*-Value**	**↑ in Gr1 (Fold-Change)**	**↑ in Gr2 (Fold-Change)**
UBP7	0.001	1.4	
HS105	0.004		2.0
DPYL2	0.006		1.2
SRSF2	0.007		1.1
FUS	0.010	1.6	
RTCB	0.012	1.4	
ANM1	0.017	1.3	
PSA1	0.020	1.2	
HNRL1	0.020		1.1
RAB5C	0.022		1.3
SYVC	0.030	1.3	
1433Z	0.032		1.2
CAH1	0.035		5.2
SPTN1	0.035	2.3	
LDHA	0.043		1.3
FLNA	0.045		1.5
ANXA6	0.046		1.3
G6PD	0.048		1.8
**Group 2 vs. Group 3**			
**Gene Name**	**ANOVA *p*-Value**	**↑ in Gr2 (Fold-Change)**	**↑ in Gr3 (Fold-Change)**
DHX9	0.000	3.4	
ATPB	0.001	6.1	
GSTK1	0.001	6.7	
AHNK	0.004	6.5	
SYNC	0.004	1.4	
TCPA	0.005	2.2	
1433G	0.007	1.3	
CH60	0.010	2.9	
VATA	0.010	2.3	
PRKDC	0.010	12.0	
TAGL2	0.011	1.7	
RPN1	0.012	1.9	
TCPH	0.013	1.7	
UB2V1	0.013		1.4
PA2G4	0.016		1.1
ROA2	0.016	1.5	
ATPA	0.018	5.9	
UBA1	0.020	1.6	
FUBP1	0.020	1.9	
TCPG	0.020	1.6	
TBB4B	0.021	4.4	
FUBP2	0.022	2.8	
PNPH	0.023		2.2
GSTO1	0.025		1.9
CAN1	0.026	1.5	
HBB	0.029		4.7
BAX	0.029	1.9	
EF2	0.030	1.4	
DDX1	0.031	3.3	
URP2	0.031	1.8	
HBA	0.032		5.4
ESTD	0.032		1.4
HBD	0.034		8.2
ACTZ	0.038	1.9	
TCPB	0.039	1.6	
CBX3	0.040		1.2
TIF1B	0.043	2.8	
PGM1	0.045		1.1
IF4A1	0.045	2.9	
CPNS1	0.047	3.5	
TCPE	0.048	1.6	
**Group 1 vs. Group 3**			
**Gene Name**	**ANOVA *p*-Value**	**↑ in Gr1 (Fold-Change)**	**↑ in Gr3 (Fold-Change)**
LA	0.001		4.1
OTUB1	0.001		2.2
CNDP2	0.001		5.3
RAN	0.001		2.5
HNRPC	0.002		4.1
HNRPQ	0.003		4.2
CH60	0.003		6.6
PRDX6	0.004		2.9
TBA1B	0.005		3.7
TERA	0.006		2.2
SET	0.006		2.2
ROA2	0.006		2.8
CAPZB	0.007	1.4	
RCC2	0.007		2.0
ECHA	0.007		4.2
ARPC4	0.007	1.3	
PTPRC	0.007	2.0	
NONO	0.008		2.5
THIO	0.009		2.9
ILF3	0.011		2.0
VIME	0.011		3.5
TALDO	0.012		2.1
LDHA	0.013		2.0
TCPH	0.013		2.3
NUCL	0.014		2.8
NAGK	0.016	1.7	
DHX9	0.016		4.1
PRDX4	0.016		1.0
TCP4	0.017		2.5
HS90A	0.018		1.9
ROA1	0.018		2.5
LDHB	0.019		2.6
EF1A3	0.020		2.4
FEN1	0.020		1.8
EF2	0.021		1.9
NPM	0.024		2.6
F10A1	0.025		2.4
1433Z	0.026		1.6
TIF1B	0.027		7.0
ESTD	0.028		2.1
HNRH1	0.029		2.4
LC7L2	0.030		2.1
TCPZ	0.030		1.7
GANAB	0.030		2.3
PGAM1	0.031	1.3	
ACTB	0.031		1.7
PARP1	0.032		2.9
RUVB2	0.032		2.1
NPS3A	0.034	1.2	
NDKB	0.034		2.2
RHOA	0.035		1.6
SFPQ	0.035		1.9
IF4A3	0.035		2.3
HNRPU	0.037		2.4
DLDH	0.039		2.6
RSSA	0.041		3.6
ROA3	0.042		2.4
G3P	0.042		2.8
RS3	0.042		4.5
FSCN1	0.044		1.0
RL40	0.046	1.2	
PDIA3	0.049		1.7
HSP7C	0.049		1.7
TSN	0.050	1.2	

## Data Availability

The data will be posted on the Open Science Framework (OSF website, https://osf.io, accessed on 18 October 2021) so that the data may be analyzed by other researchers.

## Supplementary Materials

The following are available online at https://www.mdpi.com/article/10.3390/proteomes9040042/s1. Table S1: Raw mass spec data; Table S2: Raw mass spec data; Table S3: Raw mass spec data; Table S4: Raw Luminex Data; Table S5: Protein identification data Gr1 v Gr2; Table S6: Protein identification data Gr1 v Gr3; Table S7: Protein identification data Gr2 v Gr3.
