# Supplementary material for: Identification of Protein Biomarker Signatures for Acute Myeloid Leukemia (AML) Using Both Nontargeted and Targeted Approaches
Source: Proteomes. 2021 Oct 30;9(4):42. doi: 10.3390/proteomes9040042 (PMC8628952; doi:10.3390/proteomes9040042)
Supplement: Supplementary file 1 [file proteomes-09-00042-s001.zip › proteomes-1447462-supplementary.pdf]

Supplementary

Table S1. Raw mass spec data.

LFQ intensity average Gr1	LFQ intensity average Gr2	Up Gr1 (fold)	Up Gr2 (fold)	N: ANOVA p value	T: Fasta headers
21644617.57	15204309.63	1.42		0.00148031	>sp|Q93009|UBP7_HUMAN Ubiquitin carboxyl-terminal hydrolase 7 OS=Homo sapiens GN=USP7 PE=1 SV=2
12793561.74	25805905.08		2.02	0.00444306	>sp|Q92598|HS105_HUMAN Heat shock protein 105 kDa OS=Homo sapiens GN=HSPH1 PE=1 SV=1
30463495.72	35495869.5		1.17	0.00648316	>sp|Q16555|DPYL2_HUMAN Dihydropyrimidinase-related protein 2 OS=Homo sapiens GN=DPYSL2 PE=1 SV=1
29461410.8	33291487.17		1.13	0.00737384	>sp|Q01130|SRSF2_HUMAN Serine/arginine-rich splicing factor 2 OS=Homo sapiens GN=SRSF2 PE=1 SV=4
92827047.65	58709719.15	1.58		0.0103082	>sp|P35637|FUS_HUMAN RNA-binding protein FUS OS=Homo sapiens GN=FUS PE=1 SV=1
34175553.92	23598350.28	1.45		0.0119059	>sp|Q9Y3I0|RTCB_HUMAN tRNA-splicing ligase RtcB homolog OS=Homo sapiens GN=RTCB PE=1 SV=1
40295741.1	32215519.14	1.25		0.0166749	>sp|Q99873|ANM1_HUMAN Protein arginine N-methyltransferase 1 OS=Homo sapiens GN=PRMT1 PE=1 SV=2
21075047.97	17385027.38	1.21		0.0202006	>sp|P25786|PSA1_HUMAN Proteasome subunit alpha type-1 OS=Homo sapiens GN=PSMA1 PE=1 SV=1
22797483.04	24323990.18		1.07	0.0203649	>sp|Q9BUJ2|HNRL1_HUMAN Heterogeneous nuclear ribonucleoprotein U-like protein 1 OS=Homo sapiens GN=HNRNPUL1 PE=1 SV=2
21809068.9	28647266.06		1.31	0.0218747	>sp|P51148|RAB5C_HUMAN Ras-related protein Rab-5C OS=Homo sapiens GN=RAB5C PE=1 SV=2
26584863.47	20979146.8	1.27		0.0302054	>sp|P26640|SYVC_HUMAN Valine--tRNA ligase OS=Homo sapiens GN=VARS PE=1 SV=4
484310003.2	602290316.1		1.24	0.0318372	>sp|P63104|1433Z_HUMAN 14-3-3 protein zeta/delta OS=Homo sapiens GN=YWHAZ PE=1 SV=1
145954935.4	761547718		5.22	0.0350197	>sp|P00915|CAH1_HUMAN Carbonic anhydrase 1 OS=Homo sapiens GN=CA1 PE=1 SV=2
60728863.16	26835473.85	2.26		0.0353199	>sp|Q13813|SPTN1_HUMAN Spectrin alpha chain, non-erythrocytic 1 OS=Homo sapiens GN=SPTAN1 PE=1 SV=3
389883936	513314463		1.32	0.0428754	>sp|P00338|LDHA_HUMAN L-lactate dehydrogenase A chain OS=Homo sapiens GN=LDHA PE=1 SV=2
964543265.5	1409272256		1.46	0.0446947	>sp|P21333|FLNA_HUMAN Filamin-A OS=Homo sapiens GN=FLNA PE=1 SV=4
305858031.6	394608073.8		1.29	0.0460146	>sp|P08133|ANXA6_HUMAN Annexin A6 OS=Homo sapiens GN=ANXA6 PE=1 SV=3
46788978.26	84935906.92		1.82	0.0483834	>sp|P11413|G6PD_HUMAN Glucose-6-phosphate 1-dehydrogenase OS=Homo sapiens GN=G6PD PE=1 SV=4

Table S2. Raw mass spec data.

LFQ intensity average Gr1	LFQ intensity average Gr3	Up Gr1 (fold)	Up Gr3 (fold)	N: ANOVA p value	T: Fasta headers
27989216.3	115825588.8		4.14	0.00056194	>sp|P05455|LA_HUMAN Lupus La protein OS=Homo sapiens GN=SSB PE=1 SV=2
17620651.02	38979456.62		2.21	0.000610023	>sp|Q96FW1|OTUB1_HUMAN Ubiquitin thioesterase OTUB1 OS=Homo sapiens GN=OTUB1 PE=1 SV=2
23432341.97	123795295.7		5.28	0.000993105	>sp|Q96KP4|CNDP2_HUMAN Cytosolic non-specific dipeptidase OS=Homo sapiens GN=CNDP2 PE=1 SV=2
85810447.36	217145570		2.53	0.00122911	>sp|P62826|RAN_HUMAN GTP-binding nuclear protein Ran OS=Homo sapiens GN=RAN PE=1 SV=3
99541315.27	408714160		4.11	0.00187603	>sp|P07910|HNRPC_HUMAN Heterogeneous nuclear ribonucleoproteins C1/C2 OS=Homo sapiens GN=HNRNPC PE=1 SV=4
25836497.1	109232231.9		4.23	0.00279938	>sp|O60506|HNRPQ_HUMAN Heterogeneous nuclear ribonucleoprotein Q OS=Homo sapiens GN=SYNCRIP PE=1 SV=2
50926596.61	336618814.5		6.61	0.00342468	>sp|P10809|CH60_HUMAN 60 kDa heat shock protein, mitochondrial OS=Homo sapiens GN=HSPD1 PE=1 SV=2
88870562.3	260133710		2.93	0.00410064	>sp|P30041|PRDX6_HUMAN Peroxiredoxin-6 OS=Homo sapiens GN=PRDX6 PE=1 SV=3
257475993.4	955372000		3.71	0.00491529	>sp|P68363|TBA1B_HUMAN Tubulin alpha-1B chain OS=Homo sapiens GN=TUBA1B PE=1 SV=1
157439069	351895940		2.24	0.00570874	>sp|P55072|TERA_HUMAN Transitional endoplasmic reticulum ATPase OS=Homo sapiens GN=VCP PE=1 SV=4
253272631.7	550196560		2.17	0.00608569	>sp|Q01105|SET_HUMAN Protein SET OS=Homo sapiens GN=SET PE=1 SV=3
331274406.3	940537350		2.84	0.00647353	>sp|P22626|ROA2_HUMAN Heterogeneous nuclear ribonucleoproteins A2/B1 OS=Homo sapiens GN=HNRNPA2B1 PE=1 SV=2
134035315.9	94474334.01	1.42		0.00650472	>sp|P47756|CAPZB_HUMAN F-actin-capping protein subunit beta OS=Homo sapiens GN=CAPZB PE=1 SV=4
37789633.48	75617200.34		2.00	0.00667939	>sp|Q9P258|RCC2_HUMAN Protein RCC2 OS=Homo sapiens GN=RCC2 PE=1 SV=2
31425883.71	132157901.1		4.21	0.00670399	>sp|P40939|ECHA_HUMAN Trifunctional enzyme subunit alpha, mitochondrial OS=Homo sapiens GN=HADHA PE=1 SV=2
138543856.2	109383130.3	1.27		0.00704474	>sp|P59998|ARPC4_HUMAN Actin-related protein 2/3 complex subunit 4 OS=Homo sapiens GN=ARPC4 PE=1 SV=3
69673823.57	35227649.22	1.98		0.00731217	>sp|P08575|PTPRC_HUMAN Receptor-type tyrosine-protein phosphatase C OS=Homo sapiens GN=PTPRC PE=1 SV=2
78423596.05	194113230		2.48	0.00813581	>sp|Q15233|NONO_HUMAN Non-POU domain-containing octamer-binding protein OS=Homo sapiens GN=NONO PE=1 SV=4
63043156.94	182621557.2		2.90	0.00907507	>sp|P10599|THIO_HUMAN Thioredoxin OS=Homo sapiens GN=TXN PE=1 SV=3
68000208.82	134688866.2		1.98	0.0111121	>sp|Q12906|ILF3_HUMAN Interleukin enhancer-binding factor 3 OS=Homo sapiens GN=ILF3 PE=1 SV=3
484224749.8	1677393080		3.46	0.0111418	>sp|P08670|VIME_HUMAN Vimentin OS=Homo sapiens GN=VIM PE=1 SV=4
309272399.7	656301000		2.12	0.0123849	>sp|P37837|TALDO_HUMAN Transaldolase OS=Homo sapiens GN=TALDO1 PE=1 SV=2

291835142.3	580654160		1.99	0.012542	>sp|P00338|LDHA_HUMAN L-lactate dehydrogenase A chain OS=Homo sapiens GN=LDHA PE=1 SV=2
29142878.66	67674845.62		2.32	0.01334	>sp|Q99832|TCPH_HUMAN T-complex protein 1 subunit eta OS=Homo sapiens GN=CCT7 PE=1 SV=2
304261545.1	860462700		2.83	0.0140139	>sp|P19338|NUCL_HUMAN Nucleolin OS=Homo sapiens GN=NCL PE=1 SV=3
60097496.09	35953156.78	1.67		0.0156332	>sp|Q9UJ70|NAGK_HUMAN N-acetyl-D-glucosamine kinase OS=Homo sapiens GN=NAGK PE=1 SV=4
46544255.95	189410327.9		4.07	0.0161399	>sp|Q08211|DHX9_HUMAN ATP-dependent RNA helicase A OS=Homo sapiens GN=DHX9 PE=1 SV=4
27967759.03	29115989.68		1.04	0.0162337	>sp|Q13162|PRDX4_HUMAN Peroxiredoxin-4 OS=Homo sapiens GN=PRDX4 PE=1 SV=1
56392455.54	142312109.3		2.52	0.0170011	>sp|P53999|TCP4_HUMAN Activated RNA polymerase II transcriptional coactivator p15 OS=Homo sapiens GN=SUB1 PE=1 SV=3
418131328.3	802572960		1.92	0.0176241	>sp|P07900|HS90A_HUMAN Heat shock protein HSP 90-alpha OS=Homo sapiens GN=HSP90AA1 PE=1 SV=5
203961425.8	519626660		2.55	0.0176808	>sp|P09651|ROA1_HUMAN Heterogeneous nuclear ribonucleoprotein A1 OS=Homo sapiens GN=HNRNPA1 PE=1 SV=5
174119675.7	454287660		2.61	0.0193991	>sp|P07195|LDHB_HUMAN L-lactate dehydrogenase B chain OS=Homo sapiens GN=LDHB PE=1 SV=2
207319039	499202770		2.41	0.0195896	>sp|Q5VTE0|EF1A3_HUMAN Putative elongation factor 1-alpha-like 3 OS=Homo sapiens GN=EEF1A1P5 PE=5 SV=1
18236703.29	32924694		1.81	0.0197373	>sp|P39748|FEN1_HUMAN Flap endonuclease 1 OS=Homo sapiens GN=FEN1 PE=1 SV=1
199123016	383244220		1.92	0.021345	>sp|P13639|EF2_HUMAN Elongation factor 2 OS=Homo sapiens GN=EEF2 PE=1 SV=4
202569325.4	527571910		2.60	0.0244764	>sp|P06748|NPM_HUMAN Nucleophosmin OS=Homo sapiens GN=NPM1 PE=1 SV=2
40464288.88	98374767.66		2.43	0.0245519	>sp|P50502|F10A1_HUMAN Hsc70-interacting protein OS=Homo sapiens GN=ST13 PE=1 SV=2
396624547.9	631679750		1.59	0.0264211	>sp|P63104|1433Z_HUMAN 14-3-3 protein zeta/delta OS=Homo sapiens GN=YWHAZ PE=1 SV=1
14899971.19	103620853.3		6.95	0.0267052	>sp|Q13263|TIF1B_HUMAN Transcription intermediary factor 1-beta OS=Homo sapiens GN=TRIM28 PE=1 SV=5
38781760.07	81117161.07		2.09	0.0284753	>sp|P10768|ESTD_HUMAN S-formylglutathione hydrolase OS=Homo sapiens GN=ESD PE=1 SV=2
118182692.6	280003270		2.37	0.0290622	>sp|P31943|HNRH1_HUMAN Heterogeneous nuclear ribonucleoprotein H OS=Homo sapiens GN=HNRNPH1 PE=1 SV=4
15765405.36	33229344.87		2.11	0.0296814	>sp|Q9Y383|LC7L2_HUMAN Putative RNA-binding protein Luc7-like 2 OS=Homo sapiens GN=LUC7L2 PE=1 SV=2
28420243.64	49592052.29		1.74	0.0300935	>sp|P40227|TCPZ_HUMAN T-complex protein 1 subunit zeta OS=Homo sapiens GN=CCT6A PE=1 SV=3
121973530.6	274984790		2.25	0.030321	>sp|Q14697|GANAB_HUMAN Neutral alpha-glucosidase AB OS=Homo sapiens GN=GANAB PE=1 SV=3
571008404.4	439810950	1.30		0.0312292	>sp|P18669|PGAM1_HUMAN Phosphoglycerate mutase 1 OS=Homo sapiens GN=PGAM1 PE=1 SV=2
4580514156	7759186800		1.69	0.0313467	>sp|P60709|ACTB_HUMAN Actin, cytoplasmic 1 OS=Homo sapiens GN=ACTB PE=1 SV=1

69698139.56	200688980		2.88	0.0318483	>sp|P09874|PARP1_HUMAN Poly [ADP-ribose] polymerase 1 OS=Homo sapiens GN=PARP1 PE=1 SV=4
13987403.03	29864170.01		2.14	0.0321374	>sp|Q9Y230|RUVB2_HUMAN RuvB-like 2 OS=Homo sapiens GN=RUVBL2 PE=1 SV=3
66371127.09	55672389.84	1.19		0.0336147	>sp|Q9UFN0|NPS3A_HUMAN Protein NipSnap homolog 3A OS=Homo sapiens GN=NIPSNAP3A PE=1 SV=2
60400945.83	130641368.9		2.16	0.0341093	>sp|P22392|NDKB_HUMAN Nucleoside diphosphate kinase B OS=Homo sapiens GN=NME2 PE=1 SV=1
22503407.44	35988929.57		1.60	0.034698	>sp|P61586|RHOA_HUMAN Transforming protein RhoA OS=Homo sapiens GN=RHOA PE=1 SV=1
145306473.6	283177600		1.95	0.0349487	>sp|P23246|SFPQ_HUMAN Splicing factor, proline- and glutamine-rich OS=Homo sapiens GN=SFPQ PE=1 SV=2
11350737.7	26228066.13		2.31	0.0351966	>sp|P38919|IF4A3_HUMAN Eukaryotic initiation factor 4A-III OS=Homo sapiens GN=EIF4A3 PE=1 SV=4
84881721.98	206272690		2.43	0.0371499	>sp|Q00839|HNRPU_HUMAN Heterogeneous nuclear ribonucleoprotein U OS=Homo sapiens GN=HNRNPU PE=1 SV=6
37952247.32	99179532.29		2.61	0.0390372	>sp|P09622|DLDH_HUMAN Dihydrolipoyl dehydrogenase, mitochondrial OS=Homo sapiens GN=DLD PE=1 SV=2
26305156.96	94276004.66		3.58	0.0411292	>sp|P08865|RSSA_HUMAN 40S ribosomal protein SA OS=Homo sapiens GN=RPSA PE=1 SV=4
123585653	293751070		2.38	0.0416041	>sp|P51991|ROA3_HUMAN Heterogeneous nuclear ribonucleoprotein A3 OS=Homo sapiens GN=HNRNPA3 PE=1 SV=2
352811544.8	978950640		2.77	0.0416941	>sp|P04406|G3P_HUMAN Glyceraldehyde-3-phosphate dehydrogenase OS=Homo sapiens GN=GAPDH PE=1 SV=3
29805165.9	133543109.5		4.48	0.0421742	>sp|P23396|RS3_HUMAN 40S ribosomal protein S3 OS=Homo sapiens GN=RPS3 PE=1 SV=2
21657312.97	21958325.57		1.01	0.0442945	>sp|Q16658|FSCN1_HUMAN Fascin OS=Homo sapiens GN=FSCN1 PE=1 SV=3
667307172.5	547200440	1.22		0.0458245	>sp|P62987|RL40_HUMAN Ubiquitin-60S ribosomal protein L40 OS=Homo sapiens GN=UBA52 PE=1 SV=2
249618873	421229950		1.69	0.0490485	>sp|P30101|PDIA3_HUMAN Protein disulfide-isomerase A3 OS=Homo sapiens GN=PDIA3 PE=1 SV=4
546660567	952696560		1.74	0.0493623	>sp|P11142|HSP7C_HUMAN Heat shock cognate 71 kDa protein OS=Homo sapiens GN=HSPA8 PE=1 SV=1
61202503.14	52445205.07	1.17		0.0496246	>sp|Q15631|TSN_HUMAN Translin OS=Homo sapiens GN=TSN PE=1 SV=1

Table S3. Raw mass spec data.

LFQ intensity average Gr2	LFQ intensity average Gr3	Up Gr2 (fold)	Up Gr3 (fold)	N: ANOVA p value	T: Fasta headers
425260745.6	69158701.21	6.15		0.000734536	>sp|P06576|ATPB_HUMAN ATP synthase subunit beta, mitochondrial OS=Homo sapiens GN=ATP5B PE=1 SV=3
89924415.07	13442835.74	6.69		0.0010141	>sp|Q9Y2Q3|GSTK1_HUMAN Glutathione S-transferase kappa 1 OS=Homo sapiens GN=GSTK1 PE=1 SV=3
1502380655	231590605.2	6.49		0.00367806	>sp|Q09666|AHNK_HUMAN Neuroblast differentiation-associated protein AHNAK OS=Homo sapiens GN=AHNAK PE=1 SV=2
38964933.09	28026826.18	1.39		0.00433501	>sp|O43776|SYNC_HUMAN Asparagine--tRNA ligase, cytoplasmic OS=Homo sapiens GN=NARS PE=1 SV=1
69718905.22	31406023.08	2.22		0.00494436	>sp|P17987|TCPA_HUMAN T-complex protein 1 subunit alpha OS=Homo sapiens GN=TCP1 PE=1 SV=1
96833920.83	72436996.59	1.34		0.00739921	>sp|P61981|1433G_HUMAN 14-3-3 protein gamma OS=Homo sapiens GN=YWHAG PE=1 SV=2
309078055.3	105720027.6	2.92		0.00972875	>sp|P10809|CH60_HUMAN 60 kDa heat shock protein, mitochondrial OS=Homo sapiens GN=HSPD1 PE=1 SV=2
74917286.33	32768753.47	2.29		0.0101548	>sp|P38606|VATA_HUMAN V-type proton ATPase catalytic subunit A OS=Homo sapiens GN=ATP6V1A PE=1 SV=2
179148424.7	14955863.04	11.98		0.010329	>sp|P78527|PRKDC_HUMAN DNA-dependent protein kinase catalytic subunit OS=Homo sapiens GN=PRKDC PE=1 SV=3
559741885.2	323014383.3	1.73		0.010542	>sp|P37802|TAGL2_HUMAN Transgelin-2 OS=Homo sapiens GN=TAGLN2 PE=1 SV=3
43734471.19	23355019.24	1.87		0.011983	>sp|P04843|RPN1_HUMAN Dolichyl-diphosphooligosaccharide--protein glycosyltransferase subunit 1 OS=Homo sapiens GN=RPN1 PE=1 SV=1
79722812.19	47491133.22	1.68		0.0125269	>sp|Q99832|TCPH_HUMAN T-complex protein 1 subunit eta OS=Homo sapiens GN=CCT7 PE=1 SV=2
54397971.67	73815348.1		1.36	0.0132291	>sp|Q13404|UB2V1_HUMAN Ubiquitin-conjugating enzyme E2 variant 1 OS=Homo sapiens GN=UBE2V1 PE=1 SV=2
55017867.32	58698489.43		1.07	0.0157427	>sp|Q9UQ80|PA2G4_HUMAN Proliferation-associated protein 2G4 OS=Homo sapiens GN=PA2G4 PE=1 SV=3
733244859.5	503096066.7	1.46		0.0158424	>sp|P22626|ROA2_HUMAN Heterogeneous nuclear ribonucleoproteins A2/B1 OS=Homo sapiens GN=HNRNPA2B1 PE=1 SV=2
301342173.3	50730431.26	5.94		0.0182628	>sp|P25705|ATPA_HUMAN ATP synthase subunit alpha, mitochondrial OS=Homo sapiens GN=ATP5A1 PE=1 SV=1
318876569.2	204490033.3	1.56		0.0196012	>sp|P22314|UBA1_HUMAN Ubiquitin-like modifier-activating enzyme 1 OS=Homo sapiens GN=UBA1 PE=1 SV=3
57522666.81	30003483.4	1.92		0.0200307	>sp|Q96AE4|FUBP1_HUMAN Far upstream element-binding protein 1 OS=Homo sapiens GN=FUBP1 PE=1 SV=3
78851011.28	48029547.93	1.64		0.0202385	>sp|P49368|TCPG_HUMAN T-complex protein 1 subunit gamma OS=Homo sapiens GN=CCT3 PE=1 SV=4
536682623.2	122191461.1	4.39		0.0214392	>sp|P68371|TBB4B_HUMAN Tubulin beta-4B chain OS=Homo sapiens GN=TUBB4B PE=1 SV=1
72889167.24	25702269.69	2.84		0.0219194	>sp|Q92945|FUBP2_HUMAN Far upstream element-binding protein 2 OS=Homo sapiens GN=KHSRP PE=1 SV=4
162800643.9	360397795.7		2.21	0.0229496	>sp|P00491|PNPH_HUMAN Purine nucleoside phosphorylase OS=Homo sapiens GN=PNP PE=1 SV=2

107513842.4	205286883.3		1.91	0.0252643	>sp|P78417|GSTO1_HUMAN Glutathione S-transferase omega-1 OS=Homo sapiens GN=GSTO1 PE=1 SV=2
132113983.5	89957562.44	1.47		0.0262714	>sp|P07384|CAN1_HUMAN Calpain-1 catalytic subunit OS=Homo sapiens GN=CAPN1 PE=1 SV=1
15085839033	71638488833		4.75	0.0286061	>sp|P68871|HBB_HUMAN Hemoglobin subunit beta OS=Homo sapiens GN=HBB PE=1 SV=2
25641666.64	13828333.96	1.85		0.0287331	>sp|Q07812|BAX_HUMAN Apoptosis regulator BAX OS=Homo sapiens GN=BAX PE=1 SV=1
376534058.5	276392016.7	1.36		0.0300861	>sp|P13639|EF2_HUMAN Elongation factor 2 OS=Homo sapiens GN=EEF2 PE=1 SV=4
52214809.2	15768759.17	3.31		0.0309194	>sp|Q92499|DDX1_HUMAN ATP-dependent RNA helicase DDX1 OS=Homo sapiens GN=DDX1 PE=1 SV=2
171913243	95965872.09	1.79		0.0312378	>sp|Q86UX7|URP2_HUMAN Fermitin family homolog 3 OS=Homo sapiens GN=FERMT3 PE=1 SV=1
8531695368	45842013500		5.37	0.0315205	>sp|P69905|HBA_HUMAN Hemoglobin subunit alpha OS=Homo sapiens GN=HBA1 PE=1 SV=2
63409668.94	86973938.02		1.37	0.031608	>sp|P10768|ESTD_HUMAN S-formylglutathione hydrolase OS=Homo sapiens GN=ESD PE=1 SV=2
401658839.4	3288369300		8.19	0.0343655	>sp|P02042|HBD_HUMAN Hemoglobin subunit delta OS=Homo sapiens GN=HBD PE=1 SV=2
34033177.34	17923879.29	1.90		0.0383736	>sp|P61163|ACTZ_HUMAN Alpha-centractin OS=Homo sapiens GN=ACTR1A PE=1 SV=1
118057641	72219579.68	1.63		0.0386622	>sp|P78371|TCPB_HUMAN T-complex protein 1 subunit beta OS=Homo sapiens GN=CCT2 PE=1 SV=4
55577320.87	68736095.37		1.24	0.0399822	>sp|Q13185|CBX3_HUMAN Chromobox protein homolog 3 OS=Homo sapiens GN=CBX3 PE=1 SV=4
82746746.81	29827692.27	2.77		0.0433539	>sp|Q13263|TIF1B_HUMAN Transcription intermediary factor 1-beta OS=Homo sapiens GN=TRIM28 PE=1 SV=5
33693163.91	38373060.48		1.14	0.0449425	>sp|P36871|PGM1_HUMAN Phosphoglucomutase-1 OS=Homo sapiens GN=PGM1 PE=1 SV=3
179180685.8	60889931.64	2.94		0.0454005	>sp|P60842|IF4A1_HUMAN Eukaryotic initiation factor 4A-I OS=Homo sapiens GN=EIF4A1 PE=1 SV=1
50933159.54	14614229.35	3.49		0.0469324	>sp|P04632|CPNS1_HUMAN Calpain small subunit 1 OS=Homo sapiens GN=CAPNS1 PE=1 SV=1
62647568.81	38141045.34	1.64		0.0476984	>sp|P48643|TCPE_HUMAN T-complex protein 1 subunit epsilon OS=Homo sapiens GN=CCT5 PE=1 SV=1

Table S4. Raw Luminex Data.

**SDF-1α1β**			
Labels	Group 1	Group 2	Group 3
Min	590.9366	1378.852	590.9366
Q1	1280.363	4136.556	4530.514
Median	2363.746	10045.92	9258.006
Q3	6007.855	16743.2	13985.5
Max	11621.75	43138.37	29743.81
IQR	4727.492	12606.65	9454.985
**IL-1α**			
Labels	Group 1	Group 2	Group 3
Min	46.48854	29.7048	29.7048
Q1	72.89928	52.07111	50.67546
Median	85.98344	56.86243	52.07111
Q3	99.33722	68.5379	62.97241
Max	118.4796	74.97141	172.9529
IQR	26.43793	16.46679	12.29695
**IL-17A**			
Labels	Group 1	Group 2	Group 3
Min	0.963388	0.963388	0.963388
Q1	11.07445	16.27177	6.501709
Median	30.33818	37.13759	6.501709
Q3	33.83835	54.75424	15.27789
Max	47.19422	62.87024	23.06602
IQR	22.7639	38.48247	8.776182
**IL-1RA**			
Labels	Group 1	Group 2	Group 3
Min	11.50604	27.68707	11.50604
Q1	27.68707	60.46867	25.77264
Median	27.68707	99.93881	60.46867
Q3	72.33555	188.7023	80.82589
Max	120.6615	345.0315	140.5096
IQR	44.64849	128.2336	55.05324

Table S5. Protein identification data Gr1 v Gr2.

Peptides	Sequence coverage [%]	Mol. weight [kDa]	Score	Intensity	MS/MS Count	Protein ID
22	62.7	36.688	323.31	69337000000	405	sp|P00338|LDHA_HUMAN
16	65.5	28.87	323.31	47179000000	309	sp|P00915|CAH1_HUMAN
31	51.9	75.872	323.31	40374000000	599	sp|P08133|ANXA6_HUMAN
23	47.8	59.256	323.31	6949100000	188	sp|P11413|G6PD_HUMAN
112	62.5	280.74	323.31	1.3175E+11	1871	sp|P21333|FLNA_HUMAN
9	44.9	29.555	244.41	2381800000	69	sp|P25786|PSA1_HUMAN
20	22.9	140.47	142.5	3201000000	135	sp|P26640|SYVC_HUMAN
6	14.3	53.425	96.932	9115400000	160	sp|P35637|FUS_HUMAN
7	48.6	23.482	114.73	2576200000	85	sp|P51148|RAB5C_HUMAN
16	66.9	27.745	323.31	89979000000	439	sp|P63104|1433Z_HUMAN
4	21.7	25.476	131	3089400000	55	sp|Q01130|SRSF2_HUMAN
66	35.3	284.54	323.31	7183100000	208	sp|Q13813|SPTN1_HUMAN
16	48.6	62.293	212.95	3469300000	74	sp|Q16555|DPYL2_HUMAN
17	26.6	96.864	64.451	2479800000	88	sp|Q92598|HS105_HUMAN
19	24.8	128.3	113.85	3890900000	80	sp|Q93009|UBP7_HUMAN
13	33.8	41.515	77.785	4533900000	133	sp|Q99873|ANM1_HUMAN
11	18	95.737	59.284	2620300000	95	sp|Q9BUJ2|HNRL1_HUMAN
17	47.5	55.21	114.12	5014900000	86	sp|Q9Y3I0|RTCB_HUMAN

Table S6. Protein identification data Gr1 v Gr3.

Peptides	Sequence coverage [%]	Mol. weight [kDa]	Score	Intensity	MS/MS Count	Protein IDs
19	36.8	69.602	323.31	22684000000	168	sp|O60506|HNRPQ_HUMAN
22	62.7	36.688	323.31	69337000000	405	sp|P00338|LDHA_HUMAN
17	65.7	36.053	323.31	1.8795E+11	445	sp|P04406|G3P_HUMAN
25	51.7	46.836	243.11	15064000000	224	sp|P05455|LA_HUMAN
7	37.1	32.575	323.31	50920000000	202	sp|P06748|NPM_HUMAN
16	48.8	36.638	289.23	74952000000	302	sp|P07195|LDHB_HUMAN
41	54.5	84.659	323.31	61731000000	546	sp|P07900|HS90A_HUMAN
16	37.3	33.67	124.48	33462000000	306	sp|P07910|HNRPC_HUMAN
26	24.7	147.25	181.16	4073700000	202	sp|P08575|PTPRC_HUMAN
37	67.6	53.651	323.31	1.9318E+11	627	sp|P08670|VIME_HUMAN
12	43.4	32.854	312.58	11163000000	174	sp|P08865|RSSA_HUMAN
11	32.4	54.177	178.07	8730500000	119	sp|P09622|DLDH_HUMAN
18	45.7	38.746	323.31	53786000000	334	sp|P09651|ROA1_HUMAN
37	41.7	113.08	323.31	18303000000	466	sp|P09874|PARP1_HUMAN
5	36.7	14.728	56.155	41856000000	163	sp|P62987|RL40_HUMAN
3	22.9	11.737	18.391	19812000000	68	sp|P10599|THIO_HUMAN
10	63.1	31.462	133.24	7682100000	163	sp|P10768|ESTD_HUMAN
32	71.4	61.054	323.31	43969000000	365	sp|P10809|CH60_HUMAN
28	53.7	70.897	323.31	84040000000	735	sp|P11142|HSP7C_HUMAN
41	57	95.337	323.31	44415000000	560	sp|P13639|EF2_HUMAN
15	70.1	28.804	323.31	54127000000	321	sp|P18669|PGAM1_HUMAN
20	28.5	76.613	323.31	61139000000	401	sp|P19338|NUCL_HUMAN
11	75	17.298	88.652	15715000000	164	sp|P22392|NDKB_HUMAN

20	64	37.429	323.31	79031000000	347	sp|P22626|ROA2_HUMAN
24	41.9	76.149	323.31	23539000000	431	sp|P23246|SFPQ_HUMAN
13	63.8	26.688	166.13	10504000000	161	sp|P23396|RS3_HUMAN
15	71.4	25.035	274.37	34690000000	228	sp|P30041|PRDX6_HUMAN
29	57.8	56.782	323.31	79293000000	436	sp|P30101|PDIA3_HUMAN
14	41.2	49.229	323.31	23276000000	328	sp|P31943|HNRH1_HUMAN
15	40.7	37.54	129.58	46882000000	301	sp|P37837|TALDO_HUMAN
19	45.7	46.871	55.937	3554600000	85	sp|P38919|IF4A3_HUMAN
9	29.7	42.592	33.135	3476600000	81	sp|P39748|FEN1_HUMAN
17	43.5	58.024	202.15	10635000000	194	sp|P40227|TCPZ_HUMAN
24	44.7	82.999	323.31	29054000000	299	sp|P40939|ECHA_HUMAN
14	50.5	31.35	276.94	10121000000	232	sp|P47756|CAPZB_HUMAN
7	23.6	41.331	36.722	5481900000	146	sp|P50502|F10A1_HUMAN
16	40.5	39.594	319.03	24148000000	397	sp|P51991|ROA3_HUMAN
5	37.8	14.395	28.622	9580500000	136	sp|P53999|TCP4_HUMAN
35	55.2	89.321	323.31	43333000000	514	sp|P55072|TERA_HUMAN
9	60.7	19.667	112.03	12780000000	133	sp|P59998|ARPC4_HUMAN
22	74.1	41.736	323.31	6.5149E+11	908	sp|P60709|ACTB_HUMAN
7	50.8	21.768	151.18	3710500000	82	sp|P61586|RHOA_HUMAN
10	42.6	24.423	204.38	15360000000	139	sp|P62826|RAN_HUMAN
16	66.9	27.745	323.31	89979000000	439	sp|P63104|1433Z_HUMAN
18	51.1	50.184	323.31	72420000000	320	sp|Q5VTE0|EF1A3_HUMAN
20	68.1	50.151	323.31	79289000000	378	sp|P68363|TBA1B_HUMAN
24	35.5	90.583	323.31	25720000000	318	sp|Q00839|HNRPU_HUMAN
10	52.1	33.488	323.31	31816000000	235	sp|Q01105|SET_HUMAN
33	32.3	140.96	323.31	26562000000	393	sp|Q08211|DHX9_HUMAN
22	28	95.337	323.31	16457000000	284	sp|Q12906|ILF3_HUMAN
7	35.8	30.54	95.265	3397400000	57	sp|Q13162|PRDX4_HUMAN
22	44.3	88.549	323.31	8939000000	181	sp|Q13263|TIF1B_HUMAN
30	40.7	106.87	323.31	25430000000	487	sp|Q14697|GANAB_HUMAN
18	42.5	54.231	228.67	12669000000	290	sp|Q15233|NONO_HUMAN
11	43	26.183	235.48	7081800000	123	sp|Q15631|TSN_HUMAN
14	41.4	54.529	107.77	2179000000	95	sp|Q16658|FSCN1_HUMAN
9	45.4	31.284	199.35	7136300000	93	sp|Q96FW1|OTUB1_HUMAN
16	48	52.878	137.84	9173000000	210	sp|Q96KP4|CNDP2_HUMAN
17	47.5	59.366	198.88	7158400000	186	sp|Q99832|TCPH_HUMAN
17	52.3	56.084	317.26	7763100000	179	sp|Q9P258|RCC2_HUMAN
11	62.3	28.466	70.193	3347200000	82	sp|Q9UFN0|NPS3A_HUMAN
14	57.6	37.375	177.48	6004600000	160	sp|Q9UJ70|NAGK_HUMAN
17	41.3	51.156	94.239	3570400000	117	sp|Q9Y230|RUVB2_HUMAN
9	27.8	46.513	65.335	2391000000	98	sp|Q9Y383|LC7L2_HUMAN

Table S7. Protein identification data Gr2 v Gr3.

Peptides	Sequence coverage [%]	Mol. weight [kDa]	Score	Intensity	MS/MS Count	Protein IDs
12	26.5	62.942	90.161	3196100000	104	sp|O43776|SYNC_HUMAN
17	72	32.118	323.31	19128000000	321	sp|P00491|PNPH_HUMAN
15	93.2	16.055	165.4	46038000000	221	sp|P02042|HBD_HUMAN
11	59	28.315	323.31	4009500000	95	sp|P04632|CPNS1_HUMAN
18	38.6	68.569	165.85	4968600000	145	sp|P04843|RPN1_HUMAN
22	67.9	56.559	323.31	39134000000	299	sp|P06576|ATPB_HUMAN
29	52.9	81.889	323.31	11169000000	289	sp|P07384|CAN1_HUMAN
10	63.1	31.462	133.24	7682100000	163	sp|P10768|ESTD_HUMAN
32	71.4	61.054	323.31	43969000000	365	sp|P10809|CH60_HUMAN
41	57	95.337	323.31	44415000000	560	sp|P13639|EF2_HUMAN
23	51.3	60.343	210.64	7586400000	162	sp|P17987|TCPA_HUMAN
34	49	117.85	323.31	32213000000	430	sp|P22314|UBA1_HUMAN
20	64	37.429	323.31	79031000000	347	sp|P22626|ROA2_HUMAN
22	49.4	59.75	323.31	28886000000	216	sp|P25705|ATPA_HUMAN
15	32.9	61.448	76.042	2756600000	126	sp|P36871|PGM1_HUMAN
12	75.9	22.391	323.31	65127000000	207	sp|P37802|TAGL2_HUMAN
18	41.5	68.303	323.31	7636000000	207	sp|P38606|VATA_HUMAN
24	49.4	59.67	280.38	7980300000	197	sp|P48643|TCPE_HUMAN
21	46.8	60.533	295.06	10238000000	230	sp|P49368|TCPG_HUMAN
23	55.2	46.153	285.89	19903000000	226	sp|P60842|IF4A1_HUMAN
6	28.7	42.613	80.538	2535700000	64	sp|P61163|ACTZ_HUMAN
11	56.3	28.302	224.91	15341000000	169	sp|P61981|1433G_HUMAN
20	63.1	49.83	323.31	80137000000	251	sp|P68371|TBB4B_HUMAN
20	97.3	15.998	323.31	1.5707E+12	1266	sp|P68871|HBB_HUMAN
14	78.9	15.257	323.31	1.068E+12	804	sp|P69905|HBA_HUMAN
22	61.1	57.488	323.31	13820000000	238	sp|P78371|TCPB_HUMAN
15	59.8	27.566	172.89	9665000000	272	sp|P78417|GSTO1_HUMAN
89	26.8	469.08	323.31	27833000000	502	sp|P78527|PRKDC_HUMAN
6	42.7	21.184	78.351	2164400000	76	sp|Q07812|BAX_HUMAN
33	32.3	140.96	323.31	26562000000	393	sp|Q08211|DHX9_HUMAN
184	58.2	629.09	323.31	1.0935E+11	1517	sp|Q09666|AHNK_HUMAN
7	45.9	20.811	68.487	9713300000	119	sp|Q13185|CBX3_HUMAN
22	44.3	88.549	323.31	8939000000	181	sp|Q13263|TIF1B_HUMAN
7	57.8	16.495	114.51	4271900000	111	sp|Q13404|UB2V1_HUMAN

25	48.3	75.952	323.31	15320000000	300	sp|Q86UX7|URP2_HUMAN
22	39.3	82.431	323.31	9363300000	127	sp|Q92499|DDX1_HUMAN
18	37.3	73.114	243.43	8623300000	190	sp|Q92945|FUBP2_HUMAN
20	40.5	67.56	218.79	5938100000	161	sp|Q96AE4|FUBP1_HUMAN
17	47.5	59.366	198.88	7158400000	186	sp|Q99832|TCPH_HUMAN
16	49.7	43.786	74.179	6558800000	176	sp|Q9UQ80|PA2G4_HUMAN
7	38.1	25.497	30.538	5833800000	140	sp|Q9Y2Q3|GSTK1_HUMAN
